# Neurosymbolic Systems of Perception and Cognition: The Role of Attention

**DOI:** 10.3389/fpsyg.2022.806397

**Published:** 2022-05-20

**Authors:** Hugo Latapie, Ozkan Kilic, Kristinn R. Thórisson, Pei Wang, Patrick Hammer

**Affiliations:** ^1^Emerging Technologies and Incubation, Cisco Systems, San Jose, CA, United States; ^2^Icelandic Institute for Intelligent Machines and Department of Computer Science, Reykjavik University, Reykjavik, Iceland; ^3^Department of Computer and Information Sciences, Temple University, Philadelphia, PA, United States; ^4^Center for Digital Futures, KTH Royal Institute of Technology and Stockholm University, Stockholm, Sweden

**Keywords:** artificial intelligence, cognitive architecture, levels of abstraction, neurosymbolic models, systems of thinking, thalamocortical loop

## Abstract

A cognitive architecture aimed at cumulative learning must provide the necessary information and control structures to allow agents to learn incrementally and autonomously from their experience. This involves managing an agent's goals as well as continuously relating sensory information to these in its perception-cognition information processing stack. The more varied the environment of a learning agent is, the more general and flexible must be these mechanisms to handle a wider variety of relevant patterns, tasks, and goal structures. While many researchers agree that information at different levels of abstraction likely differs in its makeup and structure and processing mechanisms, agreement on the particulars of such differences is not generally shared in the research community. A dual processing architecture (often referred to as *System-1* and *System-2)* has been proposed as a model of cognitive processing, and they are often considered as responsible for low- and high-level information, respectively. We posit that cognition is not binary in this way and that knowledge at *any* level of abstraction involves what we refer to as *neurosymbolic* information, meaning that data at both high and low levels must contain *both* symbolic and subsymbolic information. Further, we argue that the main differentiating factor between the processing of high and low levels of data abstraction can be largely attributed to the nature of the involved attention mechanisms. We describe the key arguments behind this view and review relevant evidence from the literature.

## 1. Introduction

Cognitive architectures aim to capture the information and control structures necessary to create autonomous learning agents. The sensory modalities of artificially intelligent (AI) agents operating in physical environments must measure relevant information at relatively low levels of detail, commensurate with the agent's intended tasks. Self-supervised learning makes additional requirements on the ability of an agent to dynamically and continuously relate a wide variety of sensory information to high-level goals of tasks. The more general an agent's learning is, the larger a part of its perception-cognition “information stack” must capture the necessary flexibility to accommodate a wide variety of patterns, plans, tasks, and goal structures. Low levels of cognition (close to the perceptual senses) seem to quickly generate and use predictions to generalize across similar problems. This is a key responsibility of a sensory system because low-latency predictions (i.e., those that the agent can act quickly on) are vital for survival in a rapidly changing world. Natural intelligence has several outstanding skills that Deep Learning does not have. Two of these, as pointed out by e.g., Bengio et al. (2021), are that (a) it does not require thousands of samples to learn, and (b) it can cope with out-of-order (OOD) samples. As detailed by e.g., Thórisson et al. (2019), another equally important shortcoming is that Deep Learning does not handle learning after the system leaves the laboratory—i.e., cumulative learning—in part because it does not harbor any means to verify newly acquired information *autonomously*. Such skills require not only perception processes that categorize the sensory data dynamically so that the lower levels can recognize “familiar” situations by reconfiguring known pieces and trigger higher-level cognition in the case of surprises, but also the reasoning to evaluate the new knowledge that has been thus produced. Whenever high-level cognition solves a new problem, the coordination allows the new knowledge to modify and improve the lower levels for similar future situations, which also means that both systems have access to long-term memory. Architectures addressing both sensory- and planning-levels of cognition are as of yet few and far between.

While general agreement exists in the research community that information at different levels of abstraction likely differs in makeup and structure, agreement on these differences—and thus the particulars of the required architecture and processes involved—is not widely shared. It is sometimes assumed that lower levels of abstraction are subsymbolic^1^ and higher levels symbolic, which has led some researchers to the idea that Deep Learning models are analogous to perceptual mechanisms while higher levels involve rule-based reasoning skills due to a symbolic nature, and according to e.g., Kahneman (2011), is the only system that can use language. This view has been adopted in some AI research, where “subsymbolic” processing are classified as System-1 processes, while higher-level and “symbolic” processing is considered belonging to System-2 (c.f. Smolensky, 1988; Sloman, 1996; Strack and Deutsch, 2004; Kahneman, 2011). According to this view, artificial neural networks, including Deep Learning, are System-1 processes; rule-based systems are System-2 processes (see Bengio, 2019; Bengio et al., 2021 for discussion). Similarly, James (1890) proposed that the mind has two mechanisms of thought, one which handled reasoning and another which was associative. We posit instead that cognition is not binary in this way at all, and that *any* level of abstraction involves processes operating on what might be called “*neurosymbolic”* knowledge, meaning that data at both high and low levels must accommodate *both* symbolic and subsymbolic information^2^ Further, we argue that a major differentiating factor between the processing of high and low levels of data abstraction can be largely attributed to the nature of the involved *attention mechanisms*.

More than a century ago, James (1890) defined attention as “taking possession by the mind, in clear and vivid form, of one out of what may seem several simultaneously possible objects or trains of thought...It implies withdrawal from some things in order to deal effectively with others.” We consider attention to consist of a (potentially large) set of processes whose role consists in steering the available resources of a cognitive system, from moment to moment, including (but not limited to) its short-term focus, goal pursuit, sensory control, deliberate memorization, memory retrieval, selection of sensory data, and many other subconscious control mechanisms that we can only hypothesize at this point and thus have no names for. Low-level cognition, like perception, is characterized by a relatively high-speed, distributed (“multi-threaded”), subconscious^3^ attention control, while higher-level cognition seems more “single-threaded,” and relatively slower. When people introspect, our conscious threads of attention seem to consist primarily of the latter, while much of our low-level perceptions are subconscious and under the control of autonomous attention mechanisms (see Koch and Tsuchiya, 2006; Sumner et al., 2006; Marchetti, 2011 for evidence and discussion about decoupling attention from conscious introspection). Low-level perception and cognitive operations may reflect autonomous access to long-term memory through subconscious attention mechanisms, while higher-level operation may involve the recruitment of deliberate (introspectively-accessible) cognitive control, working memory, and focused attention (Papaioannou et al., 2021).

Two separate issues in the System-1/System-2 discussion are often confused: (1) Knowledge representation and (2) information processing. The first is the (by now, familiar) “symbolic vs. subsymbolic” distinction, while the second involves the “automatic vs. controlled” distinction. Not only are these two distinctly different, they are also not perfectly aligned; while subsymbolic knowledge may be more often processed “automatically” and symbolic knowledge seem generally more accessible through voluntary control and introspection, this mapping cannot be taken as given. A classic example is skill learning like riding a bike, which starts as a controlled process, and gradually becomes automatic with increased training. On the whole this process is largely subsymbolic, with hardly anything but the top-level goals introspectively accessible to the learner of bicycle-riding (“I want to ride this bicycle without falling or crashing into things”). Though we acknowledge the above differences, in this article our focus is on the relations and correlations between these two distinctions. Given the complexity of the project and related experiments mentioned in the following sections, this article cannot fully describe the work in detail. It only covers certain aspects of the project within the scope of *attention* suitable for a general audience.

## 2. Related Work and Attention's Role in Cognition

The sharp distinction between two hypothesized systems that some AI researchers have interpreted dual-process theory to entail (cf. Posner, 2020) doesn't seem very convincing when we look at the dependencies between the necessary levels of processing. For instance, it has been demonstrated time and again (cf. Spivey et al., 2013) that expectations created verbally (“System-2 information”) have a significant influence on low-level behavior like eye movements (“System-1 information”). It is not obvious why—or how—two sharply separated control systems would be the best—or even a good—way to achieve a tight coupling between levels thus demonstrated, as has been noted by other authors (cf. Houwer, 2019). Until more direct evidence is collected for the hypothesis that there really *are* two systems (as opposed to three, four, fifty, or indeed a continuity), it is a fairly straight forward task to fit the available evidence onto that theory (cf. Strack and Deutsch, 2004). In the context of AI, more direct evidence would include a demonstration of an implemented control scheme that produced some of the same key properties as human cognition from first principles.

We would expect high-level (abstract) and low-level (perceptual/concrete) cognition to work in coordination, not competition, after millions of years of evolution. Rather than implementing a (strict, or semi-strict) pipeline structure between S1 and S2, where only data would go upstream (from S1 to S2) and only control downstream (from S2 to S1; cf. Evans and Elqayam, 2007; Evans and Stanovich, 2013; Keren, 2013; Monteiro and Norman, 2013), we hypothesize high-level and low-level cognition to be coupled through a two-way control-and-data communication, as demonstrated in numerous experiments (see Xu et al., 2020 review article on cross-modal processing between high- and low- level cognition). In other words, the low-level cognition does not solely work under control of the high-level one; rather, the two levels cooperate to optimize resource utilization through joint control.

Through the evolution of the human brain, some evidence seems to indicate that language-based conceptual representations replaced sensory-based compositional concepts, explaining the slower reaction times in humans than other mammals, e.g., chimpanzees (see for instance; Martin et al., 2014). However, this replacement may have pushed the boundaries of human higher-level cognition by allowing complex propositional representations and mental simulations. While animals do not demonstrate propositional properties of human language, researchers have found some recursion in birdsong (Gentner et al., 2006) and in syntax among bonobos (Clay and Zuberbühler, 2011). Moreover, Camp (2009) found evidence that some animals think in compositional representational systems. In other words, animals seem to lack propositional thought, but they have compositional conceptual thought, which is mostly based on integrated multisensory data. Since animals appear to have symbol-like mental representations, these findings indicate that their lower levels can be neurosymbolic. Evidence for this can be found in a significant number studies from the animal-cognition literature (for review, see Brannon, 2005; Diester and Nieder, 2007; Hauser et al., 2007; Hubbard et al., 2008; Camp, 2009).

Among the processes of key importance in skill learning, to continue with that example, is attention; a major cognitive difference between a skilled bike rider and a learner of bike-riding is what they pay attention to: The knowledgeable rider pays keen attention to the tilt angle and speed of the bicycle, responding by changing the angle of the steering wheel dynamically, in a non-linear relationship. Capable as they may already be of turning the front wheel to any desired angle, a learner is prone to fall over in large part because they don't know what to pay attention to. This is why one of the few obviously useful tips that a teacher of bicycle-riding can give a learner is to “always turn the front wheel in the direction you are falling.”

Kahneman (1973) sees attention as a pool of resources which allows different process to share cognitive capabilities and posits a System-1 that is fast, intrinsic, autonomous, emotional, parallel, and a System-2 that is slower, deliberate, conscious, and serial (Kahneman, 2011). For example, driving a car on an empty road (with no unexpected events), recognizing your mother's voice, and calculating 2+2, mostly involve System-1, whereas counting the number of people with eyeglasses in a meeting, recalling and dialing your significant other's phone number, calculating 13 × 17, and filling out a tax form depend on System-2. Kahneman's System-1 is good at making quick predictions because it constantly models similar situations based on experience. It should be noted that “experience” in this context relates to the process of learning, and its transfer—i.e., generalization and adaptation—which presumably relies heavily on higher-level cognition (and should thus be part of System-2). Learning achieved in conceptual symbolic space can be projected to subsymbolic space. In other words, since symbolic and subsymbolic spaces are in constant interaction, acquired knowledge in symbolic space has correspondences in subsymbolic space. This allows System-1 to start quickly using the projections of the knowledge, even based on System-2 experience.

Several fMRI studies support the idea that sensory-specific areas, such as thalamus, may be involved in multi-sensory stimulus integrations (Miller and D'Esposito, 2005; Noesselt et al., 2007; Werner and Noppeney, 2010), which are symbolic representations in nature. Sensory-specific brain regions are considered to be networks specialized in subsymbolic data that originates from the outside world and different body parts. Thalamo-cortical oscillation is known as a synchronization mechanism or temporal binding between different cortical regions (Llinas, 2002). However, recent evidence shows that the thalamus, previously assumed to be responsible only for relaying sensory impulses from body receptors to the cerebral cortex, can actually integrate these low-level impulses (Tyll et al., 2011; Sampathkumar et al., 2021). In other words, in the thalamus there are sensory-based integrations, and they are essential in sustaining cortical cognitive functions.

Wolff and Vann (2019) use the term “cognitive thalamus” to describe a gateway to mental representations because recent findings support the idea that thalamocortical and corticothalamic pathways may play complementary but dissociable cognitive roles (see Bolkan et al., 2017; Alcaraz et al., 2018). More specifically, the thalamocortical pathway (the fibers connecting thalamus to cortex region) can create and save task-related representations, not just purely sensory information, and this pathway is essential for updating cortical representations. Similarly, corticothalamic pathways seem to have two major functions: directing cognitive resources (focused attention) and contributing to learning. In a way, the thalamocortical pathway defines the world for the cortex, and the corticothalamic pathway uses attention to tell thalamus what the cortex needs from it to focus. Furthermore, a growing body of evidence shows that the thalamus plays a role in cognitive dysfunction, such as schizophrenia (Anticevic et al., 2014), Down's syndrome (Perry et al., 2018), drug addiction (Balleine and Leung, 2015), and ADHD (Hua et al., 2021). These discoveries support other recent findings about the role of the thalamus in cognition *via* the thalamocortical loop. The thalamus, a structure proficient in using and integrating subsymbolic data actively, describes the world for the cortex by contributing to the symbolic representations in it. On the other hand, the cortex uses attention to direct resources to refresh its symbolic representations from the subsymbolic space. In Non-Axiomatic Reasoning System (NARS; Wang, 2006) attention has the role of allocating processing power for producing and scheduling inference steps, whereby inferences can compose new representation from existing components, seek out new ones, and update the strength of existing relationships *via* knowledge revision. This control also leads to a refreshing of representations in a certain sense, as the system will utilize the representations which are most reliable and switch to alternatives if some of them turn out to be unreliable.

In the Auto-catalytic Endogenous Reflective Architecture (AERA) attention is implemented as system-permeating control of computational/cognitive resources at very fine-grain levels of processing, bounded by goals at one end and the current situation at the other (cf. Helgason et al., 2013; Nivel et al., 2015). Studies on multitasking in humans have shown that a degree of parallelism among multiple tasks is more likely if the tasks involve different data modalities, such as linguistic and tactile. Low-level attention continuously monitors both mind and the outside world and assesses situations (i.e., relates it to active goals and plans) with little or no effort, through its access to long-term memory and the sensory information. Surprises and threats and detected early in the perceptual stream, while plans and questions are handled at higher levels of abstraction, triggering higher levels of processing, which also provide a top-down control of attention and reasoning. Theoretical foundations and design features including the attention control mechanism of AERA can be fund in the detailed technical reports (cf. Thórisson, 2009; Nivel et al., 2013).

In contrast to so-called “attention” mechanisms in artificial neural networks (which are for the most part rather narrow interpretations of resource control in general), mental resources (processing power and storage in computer systems) are explicitly distributed, whereby filtering of input for useful input patterns is just a special case. Another aspect is priming for related information by activating it, which is not limited to currently perceived information but can integrate long-term memory content rather than just content of a sliding window (as in Transformers) of recent stimuli in input space.

## 3. A Neurosymbolic Architecture as Systems of Thinking

The idea of combining symbolic and sub-symbolic approaches, also known as the neurosymbolic approach, is not new. Many researchers are working on integrated neural-symbolic systems which translate symbolic knowledge into neural networks (or the other way around), because symbols, relations, and rules should have counterparts in the sub-symbolic space. Moreover, the neurosymbolic network needs a symbol manipulation that also supports preservation of the structural relations between the two systems without losing the correspondences.

Currently, Deep Learning and related machine learning methods are primarily subsymbolic. Meanwhile, rule-based systems and related reasoning systems are usually strictly symbolic. We consider it possible to have a Deep Learning model that demonstrates symbolic cognition (without reasoning mechanisms) that entails the transformation of symbolic representations into subsymbolic ML/DL/statistical models. One of the costs associated with such transformation, however, is an inevitable loss of the underlying causal model which may have existed in the symbolic representation (Parisi et al., 2019). Current subsymbolic representations are exclusively correlational; information based on spurious correlation is indistinguishable from other correlations and causal direction between correlating variables is not represented and thus not separable from either of those knowledge sets.

There is an ongoing interest in bringing symbolic and abstract thinking to Deep Learning, which could enable more powerful kinds of learning. Graph neural networks with distinct nodes (Kipf et al., 2018; Steenkiste et al., 2018), transformers with discrete positional elements (Vaswani et al., 2017), and modular models with bandwidth (Goyal and Bengio, 2020) are examples of attempts in this direction. Liu et al. (2021) summarize the advantages of having discrete values (symbols) in a Deep Learning architecture. First, using symbols allows a language for inter-modular interaction and learning, whereby the meaning of symbols is not innate but determined by the relationships with others (as in Semiotics). Second, it allows reusing previously learned symbols in unseen or out-of-order situations, by reinterpreting them in a way suitable to the situation. Discretization in Deep Learning may provide systematic generalization (recombining existing concepts) but it is currently not very successful (Lake and Baroni, 2018).

**Figure 1:**
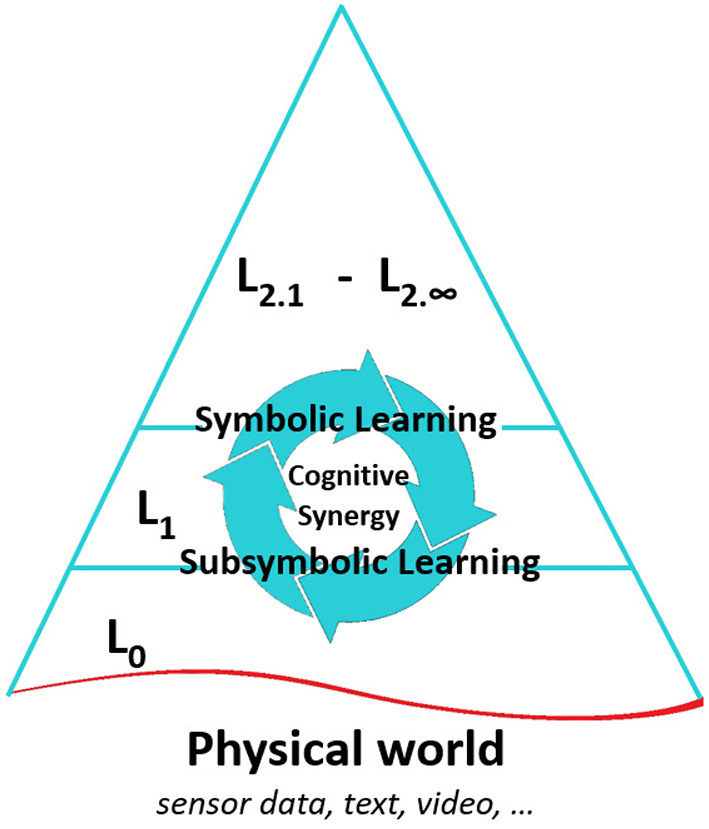
Neurosymbolic metamodel and framework for artificial general intelligence.

Current hybrid approaches attempt to combine symbolic and subsymbolic models to compensate for each other's drawbacks. However, the authors believe that there is a need for a metamodel which will accommodate hierarchical knowledge representations. Latapie et al. (2021) proposed such a model inspired by Korzybski's (1994) idea about levels of abstraction. Their model promotes cognitive synergy and metalearning, which refer to the use of different computational techniques and AGI approaches, e.g., probabilistic programming, machine learning/Deep Learning, AERA (Nivel et al., 2013; Thórisson, 2020), NARS^4^ (Wang, 2006, 2013) to enrich its knowledge and address combinatorial explosion issues. The current article extends the metamodel as a neurosymbolic architecture^5^ as in Figure 1.

In this metamodel, the levels of abstractions^6^ are marked with L. L0 is the closest to the raw data collected from various sensors. L1 contains the links between raw data and higher level abstractions. L2 corresponds to the highest integrated levels of abstraction learned through statistical learning, reasoning, and other processes. The layer L2 can have an infinite number of sub-layers since any level of abstraction in L2 can have metadata existing at an even higher level of abstraction. L* holds the high-level goals and motivations, such as self-monitoring, self-adjusting, self-repair, and the like. Similar to the previous version, the neurosymbolic metamodel is based on the assumption of insufficient knowledge and resources (Wang, 2005). The symbolic piece of the metamodel can be thought of as a knowledge graph with some additional structure that includes both a formalized means of handling anti-symmetric and symmetric relations, as well as a model of abstraction. The regions in the subsymbolic piece of the metamodel are mapped to the nodes in the symbolic system in L1. In this approach, the symbolic representations are always refreshed in a bottom-up manner.

Depending on the system's goal or subgoals, the metamodel can be readily partitioned into subgraphs using the hierarchical abstraction substructure associated with the current focus of attention. This partitioning mechanism is crucial to manage combinatorial explosion issues while enabling multiple reasoners to operate in parallel. Each partition can trigger a sub-focus of attention (sFoA), which requests subsymbolic data from System-1 or some answers from System-2. The bottom-up refreshing and the neurosymbolic mapping between regions and symbols allow the metamodel to benefit from different computational techniques (e.g., probabilistic programming, Machine Learning/Deep Learning and such) to enrich its knowledge and benefit from the ‘blessing of dimensionality' (cf. Gorban and Tyukin, 2018), also referred to as “cognitive synergy.”

**Figure 2:**
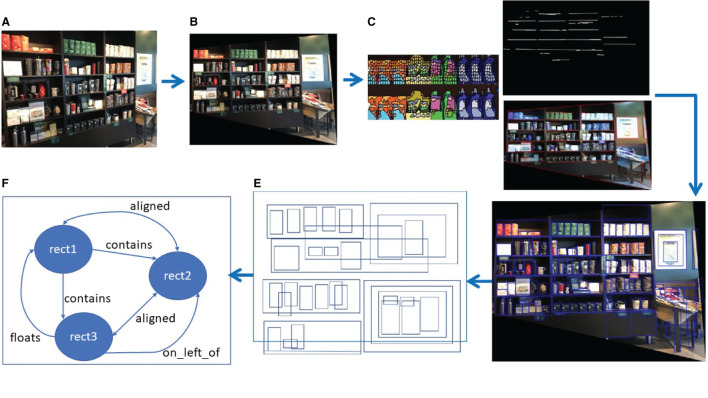
Flow of retail use case for metamodel (from Latapie et al., 2021). **(A)** Raw input from sensor data services. **(B)** Rectified input from data structuring services. **(C)** Unsupervised clustering and line detection from image processing services. **(D)** Bounding boxes from sensor data analytic services. **(E)** 2D world of rectangles. **(F)** Symbolic data and knowledge graph from spatial semantics services.

**Figure 3:**
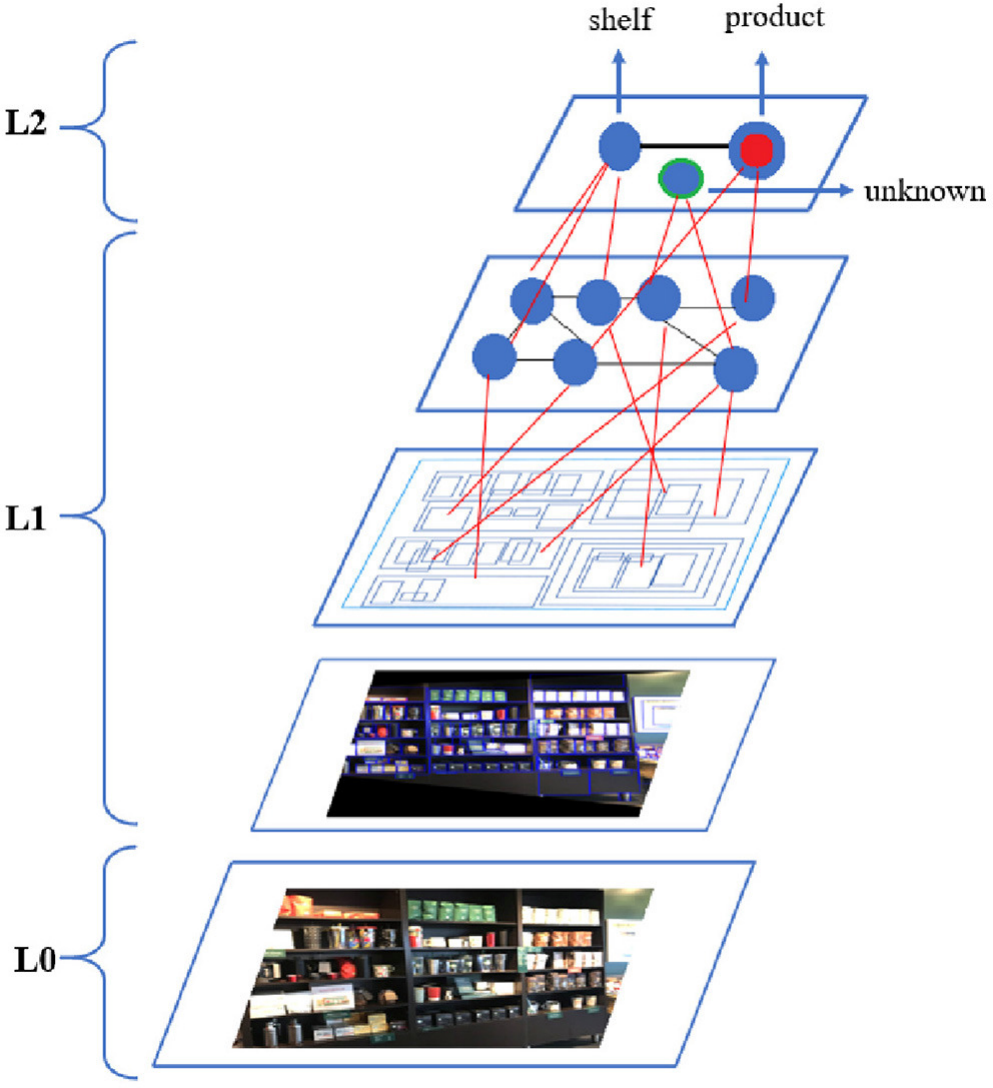
Levels of abstraction for retail use case (from Latapie et al., 2021).

A precursor to the metamodel as a neurosymbolic approach was first used by Hammer et al. (2019). This version was the first commercial implementation of a neurosymbolic AGI-aspiring^7^ approach in the smart city domain. Later, the need for use of the levels of abstraction in the metamodel became mandatory due to the combinatorial explosion issue. In other words, structural knowledge representation with the levels of abstraction became very important for partitioning the problem, process subsymbolic or symbolic information for each sub problem (focus of attention, FoA), and then combine the symbolic results in the metamodel. The metamodel with the level of abstraction was actually achieved fully in the retail domain (see Latapie et al., 2021 for details). The flow of the retail use case with the metamodel is shown in Figure 2. The example for the levels of abstraction using the results of the retail use case is shown in Figure 3. Latapie et al. (2021) emphasized that no Deep Learning model was trained with product or shelf images for the retail use case. The system used for the retail use case is solely based on representing the subsymbolic information in a world of bounding boxes with spatial semantics. The authors tested the metamodel in four different settings with and without the FoA and reported the results as in Table 1.

**Table 1:** Experimental results from retail use case using metamodel.

**Category**	**Without FoA (%)**			**With FoA (%)**		
	**Precision**	**Recall**	**f1-score**	**Precision**	**Recall**	**f1-score**
Product	80.70	29.32	52.88	96.36	99.07	97.70
Shelf	8.82	18.75	12.00	82.35	87.50	88.85
Other	36.61	89.66	52.00	96.00	82.76	88.89
Overall accuracy	**46.30** (min/max: 30.13/84.65)			**94.73** (min/max: 88.10/100.00)		

*Bold values indicate the average accuracy after 10-fold testing*.

**Figure 4:**
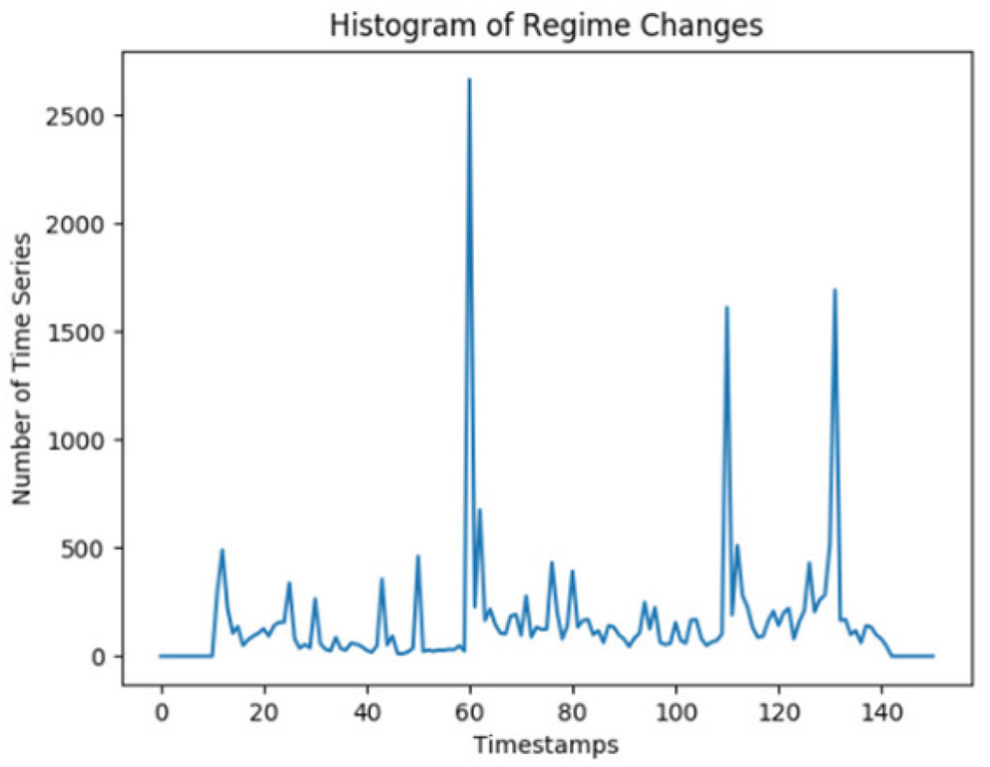
A histogram of regime changes from network telemetry data (A *port shut down* event started at the 50th timestamp and ended at the 100th).

Another use case for the metamodel is the processing of more than 200,000 time series with a total of more than 30 million individual data points. The time series are network telemetry data. For this use case, there are only two underlying assumptions: The first assumption is that the time series or a subset of them is at least weakly-related, such as time series from computer network devices. The second assumption is that when a number of time series simultaneously change their behaviors, it might indicate that an event-of-interest has happened. For detecting anomalies and finding regime change locations, Matrix Profile algorithms are used (see Yeh et al., 2016; Gharghabi et al., 2017 for Matrix Profile and Semantic Segmentation). Similar to the retail use case, millions of sensory data points are reduced to a much smaller number of events based on the semantic segmentation points. These points are used to form a histogram of regime changes as shown in Figure 4. The large *spikes* in the histogram are identified as the *candidate events-of-interest*. Then the metamodel creates a descriptive model for all time series, which allows system to downsize millions of data points into a few thousand structural actionable and explainable knowledge.

To test the metamodel with time series, we first use a subset of the Cisco Open Telemetry Data Set^8^. After being able to identify the anomalies in the data set, we create our own data sets similar to the Open Telemetry Data. For this purpose, 30 computer network events, such as memory leak, transceiver pull, port flap, port shut down, and such, are injected to a physical computer network. The system is able to identify 100% of the events with a maximum of 1 minute delay. For example, Figure 4 represents the histogram of regime changes for a *port shut down* event, which is injected at the 50th timestamp. Since the sampling rate is 6 s, 1 min later (which is at the 60th timestamp) the system detects a spike as an event-of-interest. It can take time for a single incident to display a cascading effect on multiple devices. When the injection ends at the 100th timestamp, another spike is observed within 10 timestamps, which represents a recovery behavior for the network. It should be noted that not all events necessarily mean an error has happened. Some usual activities in the network, e.g., a usual firmware update on multiple devices as events-of-no-interest, are also captured by the metamodel. The metamodel learns to classify such activities either by observing the network. Although the time series processing using the metamodel does not require any knowledge of computer networking, it can easily incorporate such features extracted by networking-specific modules, e.g., Cisco Joy,^9^ or ingest some expert knowledge defined in the symbolic world, specifically at the 2nd level of abstraction This neurosymbolic approach with the metamodel can quickly reduce the sensory data into knowledge, reason on this knowledge, and notify the network operators for remediation or trigger a self-healing protocol.

## 4. Discussion

The neurosymbolic approach presented here evolved from several independent research efforts by four core teams [NARS, AERA, OpenCog (Hart and Goertzel, 2008)], all of which are open source projects) as well as efforts at Cisco over the past 10 years focusing on hybrid state-of-the-art AI for commercial applications. This empirically-based approach to AI took off (circa 2010) with deep-learning based computer vision, augmented by well-known tracking algorithms (e.g., Kalman filtering/Hungarian algorithm). The initial hybrid architecture resulted in improved object detection and tracking functionality, but the types of errors, arguably related to weak knowledge representation and poor ability to define and learn complex behaviors, resulted in systems which did not meet our performance objectives. This initial hybrid architecture was called DFRE, Deep Fusion Reasoning Engine, which actually lacked the metamodel. In order to improve the system's ability to generalize, NARS was incorporated. The initial architecture used NARS to reason about objects and their movements in busy city intersection with trains, busses, pedestrians, and heavy traffic. This initial attempt at a commercial neurosymbolic system dramatically improved the ability of the system to generalize and learn behaviors of interest, which in this case were all related to safety. In essence the objective of the system was to raise alerts if any two moving objects either made contact or were predicted to make contact as well as to learn other dangerous behaviors such as jay walking, wrong-way driving, and such. While this system worked well as an initial prototype and is considered a success, there were early indications of potential computational scalability issues if the number of objects requiring real-time processing were to increase from the average 100 or so to say an order of magnitude more objects, such as 1,000. In order to explore this problem we then focused on a retail inventory use case that required the processing of over 1,000 objects. As expected, DFRE suffered from the predicted combinatorial explosion issues. In the retail use case, this problem was solved *via* the metamodel's abstraction hierarchy which provides a natural knowledge partitioning mechanism. This partitioning mechanism was used to address the exponential time complexity problem and convert it to a linear time complexity problem.

While NARS enabled the system to learn by reasoning in an unsupervised manner, there was a growing need in commercial applications for a principled mechanism for unsupervised learning directly from temporal data streams such as sensor data, video data, telemetry data, etc. This is the focus of AERA as well as internal Cisco project Kronos based on Matrix Profile (Yeh et al., 2016). While there is a large body of work on time series processing (FFT, Wavelets, Matrix Profile, etc.), the problem of dealing with large-scale time series and incorporating contextual knowledge to produce descriptive and predictive models with explanatory capability seems relatively unsolved at the time of this writing. In our preliminary experimentation, both AERA and Cisco's Kronos projects are demonstrating promising results. Incorporating AERA and Kronos into the hybrid architecture is expected to result in enhanced unsupervised learning and attention mechanisms directly from large-scale time series.

This evolved hybrid architecture (ML/DL/NARS/Kronos metamodel) is expected to promote cognitive synergy while preserving level of abstraction, symmetric and anti-symmetric properties of knowledge and using a bottom-up approach to refresh System-2 symbols from System-1 data integration (see Latapie et al., 2021 for details). Moreover, System-1 provides rapid responses to the outside world and activates System-2 in case of a surprise such as an emergency or other significant event that requires further analysis and potential action. System-2 uses conscious attention to request subsymbolic knowledge and sensory data from System-1, to be integrated into the levels of abstraction inspired from Korzybski's work. Korzybski's two major works (Korzybski, 1921, 1994) emphasize the importance of bottom-up knowledge. The corticothalamic and thalamocortical connections play different but complementary roles.

A balanced interplay between System-1 and System-2 is important. System-1's innate role is to ensure the many faceted health of the organism. System-2 is ideally used to help humans better contend with surprises, threats, complex situations, important goals, and achieve higher levels in Maslow's hierarchy of needs. From an AI systems perspective, contemporary Deep/Machine Learning methods (including Deep Learning) have it the other way around: Causal modeling and advanced reasoning are being solved in System 1, leveraging statistical models which can be seen as an inversion of proper thalamocortical integration.

## 5. Conclusions

While not conclusive, findings about natural intelligence from psychology, neuroscience, cognitive science, and animal cognition imply that both low-level perceptual knowledge and higher-level more abstract knowledge may be neurosymbolic. The difference between high and low levels of abstraction may be that lower levels involve a greater amount of unconscious (automatic) processing and attention, while higher levels are introspectable to a greater extent (in humans, at least) and involve conscious (i.e. steerable) attention. The neurosymbolic metamodel and framework introduced in this article for artificial general intelligence is based on these findings, and the nature of the distinction between both systems will be subject to further research. One may ask whether artificial intelligence needs to mimic natural intelligence as a key performance indicator. The answer is yes and no. No, because natural intelligence, a result of billions of years of evolution, is full of imperfections and mistakes. Yes, because it is the best way known to help organisms survive for countless generations.

Both natural and artificial intelligences can exhibit astounding generalizability, performance, ability to learn, and other important adaptive behaviors when symbolic originating attention and sub-symbolic originating attention are properly handled. Allowing one system of attention to dominate, or inverting the natural order (e.g., reasoning in the subsymbolic space or projecting symbolic space stressors into the subsymbolic space) may lead to suboptimal results for engineered systems, individuals, and societies.

## Data Availability Statement

The raw data supporting the conclusions of this article will be made available by the authors, without undue reservation.

## Author Contributions

HL and OK conceived of the presented idea and implemented the framework. HL designed the framework and the experiments. OK ran the tests and collected data. KT, PW, and PH contributed to the theoretical framework. All authors contributed to the writing of this manuscript and approved the final version.

## Conflict of Interest

The authors declare that the research was conducted in the absence of any commercial or financial relationships that could be construed as a potential conflict of interest.

## Publisher's Note

All claims expressed in this article are solely those of the authors and do not necessarily represent those of their affiliated organizations, or those of the publisher, the editors and the reviewers. Any product that may be evaluated in this article, or claim that may be made by its manufacturer, is not guaranteed or endorsed by the publisher.

## Footnotes

^1^We classify data as “subsymbolic” if it can only be manipulated through approximate similarity-mapping processes, i.e., cannot be grouped and addressed as a (named) set.

^2^By “symbolic” here we mean that the information is at the level of abstraction close to human verbal description, not that it uses “symbols” that must be interpreted or “grounded” to become meaningful.

^3^We consider “subconscious” cognitive processes the set of processes that are necessary for thought and that a mind cannot make the subject of its own cognitive processing, i.e., all its processes that it does not have direct *intropsective* access to.

^4^With open-source implementation OpenNARS at https://github.com/opennars/opennars (accessed October 20th, 2021).

^5^This architecture has a “symbolic” aspect in the sense that there are components that can be are accessed and manipulated using their identifiers. This is different from traditional Symbolic AI where a “symbol” gets its meaning by referring to an external object or event, as stated by Newell and Simon (1976).

^6^Krozybski (1994) states that knowledge is a multiordinal, hierarchical structure with varying levels of abstraction.

^7^Artificial general intelligence (AGI) is the research area closest to the original vision of the field of AI, namely, to create machines with intelligence on par with humans.

^8^ https://github.com/cisco-ie/telemetry

^9^ https://github.com/cisco/joy
